# High-Yield Expression and Purification of Scygonadin, an Antimicrobial Peptide, Using the Small Metal-Binding Protein SmbP

**DOI:** 10.3390/microorganisms12020278

**Published:** 2024-01-28

**Authors:** Jessica J. Gomez-Lugo, Nestor G. Casillas-Vega, Alma Gomez-Loredo, Isaias Balderas-Renteria, Xristo Zarate

**Affiliations:** 1 Facultad de Ciencias Quimicas, Universidad Autonoma de Nuevo Leon, Avenida Universidad s/n, Ciudad Universitaria, San Nicolas de los Garza 66455, Mexico; jessica.gomezlg@uanl.edu.mx (J.J.G.-L.); alma.gomezlr@uanl.edu.mx (A.G.-L.); isaias.balderasrn@uanl.edu.mx (I.B.-R.); 2 Departamento de Patologia Clinica, Hospital Universitario Dr. Jose Eleuterio Gonzalez, Facultad de Medicina, Universidad Autonoma de Nuevo Leon, Monterrey 64460, Mexico; ncasillasv@uanl.edu.mx; 3 Centro de Investigacion en Biotecnologia y Nanotecnologia, Facultad de Ciencias Quimicas, Universidad Autonoma de Nuevo Leon, Parque de Investigacion e Innovacion Tecnologica, Km 10 Autopista al Aeropuerto Mariano Escobedo, Apodaca 66629, Mexico

**Keywords:** scygonadin, SmbP, antimicrobial peptides, *Escherichia coli*, *Staphylococcus aureus*, immobilized metal-affinity chromatography, recombinant peptides

## Abstract

(1) Background: Producing active antimicrobial peptides with disulfide bonds in bacterial strains is challenging. The cytoplasm of *Escherichia coli* has a reducing environment, which is not favorable to the formation of disulfide bonds. Additionally, *E. coli* may express proteins as insoluble aggregates known as inclusion bodies and have proteolytic systems that can degrade recombinant peptides. Using *E. coli* strains like SHuffle and tagging the peptides with fusion proteins is a common strategy to overcome these difficulties. Still, the larger size of carrier proteins can affect the final yield of recombinant peptides. Therefore, a small fusion protein that can be purified using affinity chromatography may be an ideal strategy for producing antimicrobial peptides in *E. coli*. (2) Methods: In this study, we investigated the use of the small metal-binding protein SmbP as a fusion partner for expressing and purifying the antimicrobial peptide scygonadin in *E. coli*. Two constructs were designed: a monomer and a tandem repeat; both were tagged with SmbP at the N-terminus. The constructs were expressed in *E. coli* SHuffle T7 and purified using immobilized metal-affinity chromatography. Finally, their antimicrobial activity was determined against *Staphylococcus aureus*. (3) Results: SmbP is a remarkable fusion partner for purifying both scygonadin constructs, yielding around 20 mg for the monomer and 30 mg for the tandem repeat per 1 mL of IMAC column, reaching 95% purity. Both protein constructs demonstrated antimicrobial activity against *S. aureus* at MICs of 4 μM and 40 μM, respectively. (4) Conclusions: This study demonstrates the potential of SmbP for producing active peptides for therapeutic applications. The two scygonadin constructs in this work showed promising antimicrobial activity against *S. aureus*, suggesting they could be potential candidates for developing new antimicrobial drugs.

## 1. Introduction

The production of recombinant proteins and peptides in *Escherichia coli* has significantly benefited from fusion, or carrier proteins. They may increase protein solubility and, sometimes, allow efficient purification directly from the cell lysate using affinity chromatography [1]. Immobilized metal affinity chromatography (IMAC) is one of the most widely used protein purification methods since it is readily available and affordable; that is why the His-tag is one of the most used tags for protein purification, although it sometimes affects protein solubility [2,3].

Many proteins and peptides have disulfide bonds formed by the oxidation of sulfhydryl groups between two cysteine side chains, resulting in a covalent bond, increasing their stability and biological activity [4]. In *E. coli*, the periplasm is the only cellular space where disulfide bonds are formed due to its oxidative nature and the presence of enzymes that catalyze their formation and isomerization [5]. In contrast, the cytoplasm lacks these components and has active systems that reduce disulfide bonds, such as the glutathione reductase and thioredoxin reductase pathways [6]. Modified *E. coli* strains have been designed to circumvent the problem associated with disulfide bond formation in their cytoplasm, like SHuffle and Origami [7,8]. These strains have a total or partial interruption of one or both pathways involved in ensuring that the cytoplasm has a reducing environment. Furthermore, *E. coli* SHuffle has the disulfide isomerase DsbC responsible for forming and properly folding proteins with disulfide bonds [9].

Antimicrobial peptides have demonstrated a broad range of antimicrobial activities against bacteria and fungi; they are usually small, can be found in prokaryotic and eukaryotic organisms, and primarily target microbial membranes [10]. Antimicrobial peptides are an excellent source of biological molecules to fight pathogens in these challenging times of antibiotic resistance, and they can enhance the effectiveness of traditional antibiotics by disrupting bacterial membranes and increasing antibiotic permeability [11]. Many antimicrobial peptides have disulfide bonds in their structure, and even though it is possible to produce disulfide-bonded antimicrobial peptides in the cytoplasm of *E. coli* SHuffle or Origami, one of the most common problems during the expression of these recombinant peptides is the formation of inclusion bodies [12]. One way to solve this problem is by using fusion proteins; however, the multiple-size comparison between the fusion protein and the target peptide affects the peptide’s yield. Other strategies for producing antimicrobial peptides in *E. coli* exist; for example, the expression of recombinant proteins as multimeric tandems, which consists of copying the gene of interest two or more times to increase the gene’s transcription level and eventually improve the peptide’s final yield [13].

The small metal-binding protein (SmbP), isolated from the bacterium *Nitrosomonas europaea*, is a periplasmic protein that contains a high percentage of histidine and acidic residues that serve as ligands to bind different metal ions such as Cu(II), Mn(II), Zn(II), and Ni(II), among others, and its biology is the expulsion of excess copper ions from the cell [14]. This feature makes SmbP attractive for tagging recombinant proteins and peptides, so they can be purified using IMAC. Furthermore, SmbP increases the amount of soluble protein by decreasing the formation of inclusion bodies, and it has a molecular weight of only 10 kDa, improving the final yield of the protein or peptide of interest after tag removal [15]. Our laboratory has also proposed an alternative metal-binding protein, CusF3H+, as a carrier protein with the same advantages as SmbP for recombinant protein expression and purification in *E. coli* [16].

Scygonadin (SCY) is an anionic antibacterial peptide that was isolated from the seminal plasma of the mud crab, *Scylla serrata* [17]. It shows antibacterial activity against Gram-positive and Gram-negative bacteria like *Micrococcus luteus* and *Aeromonas hydrophila*, suggesting it targets conserved bacterial components like the membrane rather than specific receptors, potentially reducing the risk of resistance development [18]. For complete antimicrobial activity, SCY must form a disulfide bond between the Cys33 and Cys51 residues [19]. SCY’s initial interaction with the bacterial membrane involves its amphipathic nature, which is characterized by hydrophobic and hydrophilic regions. The positively charged hydrophilic segment, attributed to arginine and lysine residues, attracts negatively charged lipopolysaccharides on the outer membrane. Following membrane binding, SCY’s hydrophobic region inserts into the lipid bilayer, potentially inducing pore formation or disrupting the phospholipid arrangement. This action may result in the leakage of essential intracellular components, ultimately leading to cell death. Other studies suggest that SCY might not solely rely on membrane disruption; it could potentially interact with and disrupt vital intracellular processes like DNA synthesis or protein translation, further contributing to its antimicrobial activity [17,18,20]. Furthermore, there is speculation that SCY may work synergistically with other immune molecules in mud crabs, such as lysozyme or reactive oxygen species, enhancing the overall antimicrobial defense system [20].

Due to SCY’s appeal as a potential antimicrobial agent, particularly against multidrug-resistant bacteria, optimizing its expression and purification is crucial. Here, we applied two recombinant expression strategies: first, we tagged SCY with SmbP as a fusion protein at the N-terminus (SmbP_SCY); second, a tandem repeat (2 repeats) of SCY joined with a flexible sequence (GGGS)_3_, while using SmbP as carrier protein (SmbP_SCY FLEX). We selected *E. coli* SHuffle T7(DE3) as the expression host for proper disulfide bond formation and the enzyme enterokinase for tag removal and SCY release. Since SmbP was used for both constructs, only IMAC was necessary to obtain pure peptide from the crude lysates for in vitro antimicrobial assays against *Staphylococcus aureus*.

## 2. Materials and Methods

### 2.1. DNA Constructs

The SCY FLEX gene was synthesized by GenScript (Piscataway, NJ, USA). The plasmid we used was pET30a(+)_SmbP, which was previously constructed in our laboratory [15]. The expression strain *Escherichia coli* SHuffle T7(DE3), restriction enzymes, *Vent* DNA polymerase, and T4 DNA ligase were purchased from New England Biolabs (Ipswich, MA, USA); the enterokinase, His, bovine was purchased from GenScript.

From the SCY FLEX gene, we amplified the SCY gene using the forward primer 5′-CCATGGCTGGGCAGGCG-3′ (NcoI restriction site underlined) and the reverse primer 5′-CTCGAGTTAATAGCTCGCG-3′ (XhoI restriction site underlined). The amplification reaction was carried out using 10 ng of SCY FLEX DNA template, 60 pmoles of each primer, 1.5 μL of dNTP mix (10 mM), 10× ThermoPol reaction buffer, and 1 U of *Vent* DNA polymerase in a total volume of 50 μL. The plasmid pET30a(+)_SmbP was linearized with NcoI and XhoI. The amplified SCY DNA was digested with the same enzymes. The SCY FLEX gene was directly linearized with NcoI and XhoI. Both pET30a(+)_SmbP, SCY, and SCY FLEX were ligated with T4 DNA ligase. Ligation products were transformed into *E. coli* DH5α for posterior plasmid DNA extraction and characterization.

The pathogen *Staphylococcus aureus* used for the antimicrobial activity was supplied by the University Hospital, Department of Pathology, Clinical Microbiology Laboratory (UANL, Monterrey, Mexico).

### 2.2. Protein Expression

DNA constructs were transformed into *E. coli* SHuffle T7 Express. For small-scale expression experiments, 2 mL of Luria–Bertani (LB) broth with kanamycin (30 μg/mL) were inoculated with a single colony and incubated at 37 °C and 200 rpm until an OD_600_ between 0.4 and 0.8 was reached. Protein expression was induced with IPTG to a final concentration of 0.1 mM; cells were incubated at 17 °C or 37 °C and 220 rpm for 16 h. Cells were harvested and resuspended in 120 μL of ice-cold lysis buffer (50 mM Tris-HCl, 500 mM NaCl, pH 8.0) and lysed using 0.1 mm glass beads and vortexing for 4 min. The lysate was centrifuged for 10 min at 13,000 rpm. The supernatant was used to analyze the soluble protein content by using SDS-PAGE electrophoresis. For large-scale protein expression, SmbP_SCY and SmbP_SCY FLEX were grown in baffled flasks until OD_600_ reached 0.4–0.8, and expression was induced by adding isopropyl β-d-thiogalactoside (IPTG) up to 0.1 mM; cells were incubated at 17 °C and 220 rpm for 16 h.

### 2.3. Protein Purification

SmbP_SCY and SmbP_SCY FLEX cells were harvested by centrifugation at 4 °C and 8000 rpm for 20 min. Cells were resuspended in ice-cold lysis buffer and lysed using 0.1 mm glass beads and vortexing. The lysate was clarified by centrifugation at 4 °C and 10,000 rpm for 20 min. Purification was carried out using the ÄKTA Prime Plus FPLC System (GE Healthcare, Chicago, IL, USA). A 1 mL HiTrap IMAC FF column was equilibrated with lysis buffer; after loading the lysate, the column was washed with 10 column volumes of washing buffer (50 mM Tris-HCl, 500 mM NaCl, 5 mM imidazole, pH 8.0) and then eluted with elution buffer (50 mM Tris-HCl, 500 mM NaCl, 200 mM imidazole, pH 8.0). Elution fractions (1 mL) were collected and analyzed using sodium dodecyl sulfate-polyacrylamide gel electrophoresis (SDS-PAGE). After IMAC purification, both protein constructs were dialyzed against 50 mM Tris-HCl, pH 7.4, using the SnakeSkin dialysis membrane (Thermo Fisher Scientific, Waltham, MA, USA) and digested with enterokinase for 16 h at room temperature. Finally, a second round of IMAC chromatography was made for each protein to remove SmbP using high-affinity Ni-charged resin (GenScript, Piscataway, NJ, USA). Tag removal for final SCY or SCY FLEX purification was analyzed using SDS-PAGE; protein purity was determined using densitometry using the ImageJ software (v.2.0) [21], and protein quantity was determined using the bicinchoninic acid (BCA) method with bovine serum albumin (BSA) as standard [22].

### 2.4. Antimicrobial Activity

The broth microdilution method was applied to assess antimicrobial activity and determine the minimal inhibitory concentration (MIC) [23]. The antibacterial activity of recombinant SCY and SCY FLEX was tested at pH 7.4. *S. aureus* was cultured in LB broth and diluted in a sterile 0.85% NaCl solution with a final cell suspension of 1 × 10^5^ CFU/mL. The assay mixture consisted of 50 μL diluted purified peptide at different concentrations, 30 μL of *S. aureus* suspension, and 20 μL culture media. After 24 h of incubation at 28 °C, plates were read at 630 nm, and data were analyzed using GraphPad Prism 7 for Windows (GraphPad Software, Boston, MA, USA, www.graphpad.com, accessed on 5 June 2023), where the MIC was calculated. We conducted a Student’s *t*-test to assess for statistical differences against the negative control.

## 3. Results

### 3.1. DNA Constructs

**Figure 1:**
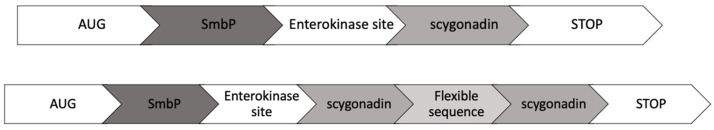
SCY and SCY FLEX expression strategies.

**Figure 2:**
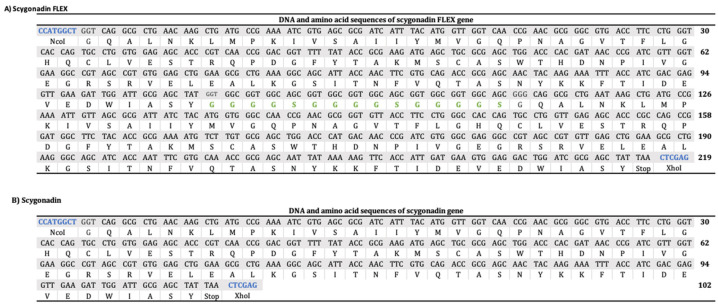
DNA and amino acid sequences for SCY FLEX (**A**) and SCY (**B**). Restriction sites are shown in blue, while flexible sequences are shown in green.

Figure 1 shows the construct design for SCY and SCY FLEX. The encoding sequence for SCY was obtained from the Database of Antimicrobial Activity and Structure of Peptides (DBAASP ID 8766), and (GGGGS)_3_ was used as a flexible sequence for SCY FLEX. We also added the NcoI and XhoI restriction sites for cloning into the plasmid pET30a(+) that already has the SmbP DNA sequence cloned using the restriction sites NdeI and KpnI; the constructs have an enterokinase cleavage site between SmbP and SCY. Figure 2 details the DNA and amino acid sequences for the SCY FLEX and SCY, respectively.

### 3.2. Protein Expression and Purification

**Figure 3:**
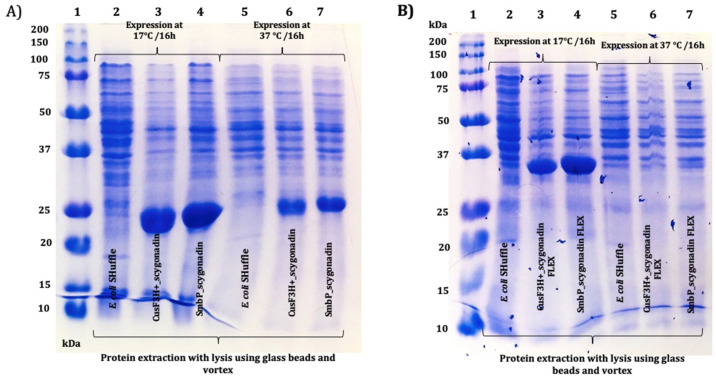
The 12% SDS-PAGE analysis of small-scale expression of SCY and SCY FLEX using two different fusion proteins and different expression temperatures in *E. coli* Shuffle: (**A**) Lane 1: protein marker; lanes 2 and 5: untransformed *E. coli* SHuffle cell lysate at 17 °C and 37 °C, respectively; lanes 3 and 6: CusF3H+_SCY (21.99 kDa) at 17 °C and 37 °C, respectively; lanes 4 and 7: SmbP_SCY (21.70 kDa) at 17 °C and 37 °C, respectively. (**B**) Lane 1: protein marker; lanes 2 and 5: untransformed *E. coli* SHuffle cell lysate at 17 °C and 37 °C, respectively; lanes 3 and 6: CusF3H+_SCY FLEX (34.19 kDa) at 17 °C and 37 °C, respectively; lanes 4 and 7: SmbP_SCY FLEX (33.9 kDa) at 17 °C and 37 °C, respectively.

After the DNA constructs were characterized, a series of small-scale expressions were performed; each recombinant protein was transformed into *E. coli* SHuffle T7(DE3), cells were incubated at 37 °C until OD_600_ reached between 0.4 and 0.8, and protein expression was induced using 0.1 M IPTG to a final concentration of 0.1 mM and incubated at 17 °C or 37 °C for 16 h. From each treatment, the protein was recovered and analyzed using SDS-PAGE. Figure 3A shows the results for SCY for the two expression temperatures while using SmbP or CusF3H+ as carried proteins (as mentioned above, the latter is an alternative fusion protein designed in our laboratory for recombinant protein expression and purification in *E. coli*). Figure 3B shows the results for SmbP_SCY FLEX and CusF3H+_SCY FLEX. Since the expression at 17 °C while using SmbP as a fusion protein showed slightly better results, we continued the experiments for each recombinant protein using these conditions.

**Figure 4:**
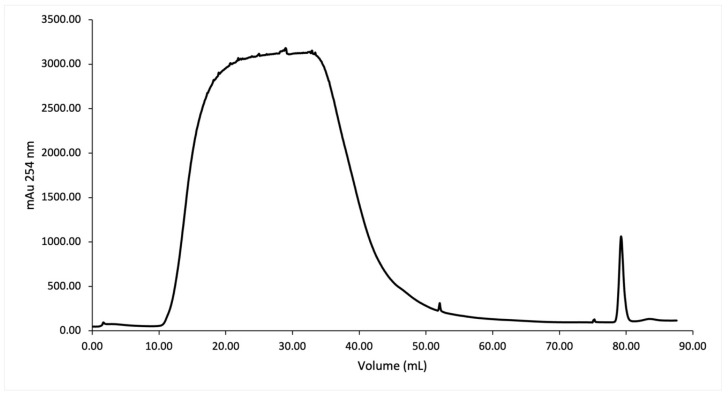
IMAC purification of SmbP_SCY using one-step elution. Chromatogram from the purification using the ÄKTA Prime Plus system with a 1 mL His-Trap FF column. The column was equilibrated with 50 mM Tris–HCl, 500 mM NaCl, pH 8; the elution was performed with 50 mM Tris–HCl, 500 mM NaCl, and 200 mM imidazole, pH 8.

**Figure 5:**
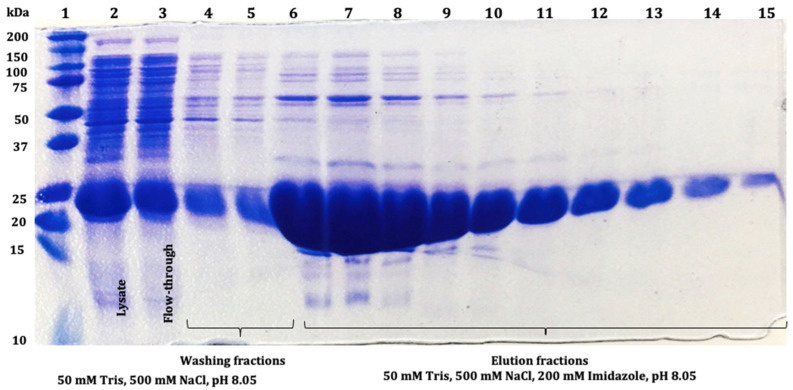
The 12% SDS-PAGE analysis of SmbP_SCY (21.70 kDa) fractions obtained after IMAC purification: lane 1: protein marker; lane 2: lysate; lane 3: flow-through; lanes 4 and 5: washing fractions; lanes 6 to 15: SmbP_SCY elution fractions.

**Figure 6:**
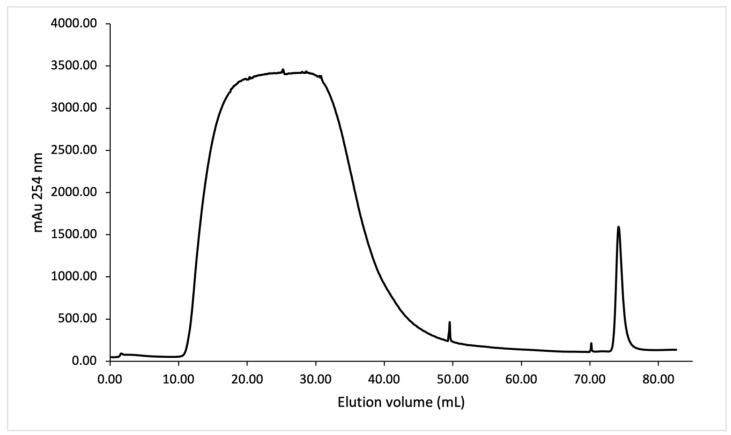
IMAC purification of SmbP_SCY FLEX (33.9 kDa) using one-step elution. Chromatogram from the purification using the ÄKTA Prime Plus system with a 1 mL His-Trap FF column. The column was equilibrated with 50 mM Tris–HCl, 500 mM NaCl, pH 8; the elution was performed with 50 mM Tris–HCl, 500 mM NaCl, and 200 mM imidazole, pH 8.

**Figure 7:**
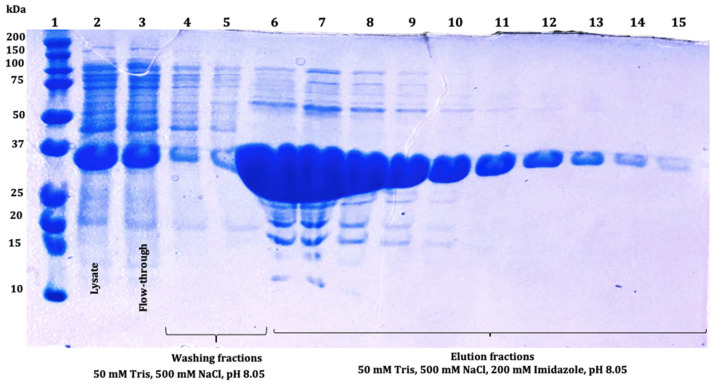
The 12% SDS-PAGE analysis of SmbP_SCY FLEX (33.9 kDa) fractions obtained after IMAC purification: lane 1: protein marker; lane 2: lysate; lane 3: flow-through; lanes 4 and 5: washing fractions; lanes 6 to 15: SmbP_SCY FLEX elution fractions.

SmbP_SCY and SmbP_SCY FLEX expressions were scaled up to one liter using the above conditions and purified using IMAC. Figure 4 shows the chromatogram for SmbP_SCY purification; the lysate, flow-through, washing, and elution fractions were analyzed using electrophoresis as shown in Figure 5. Figure 6 and Figure 7 show the chromatogram and electrophoretic analysis of SmbP_SCY FLEX.

**Figure 8:**
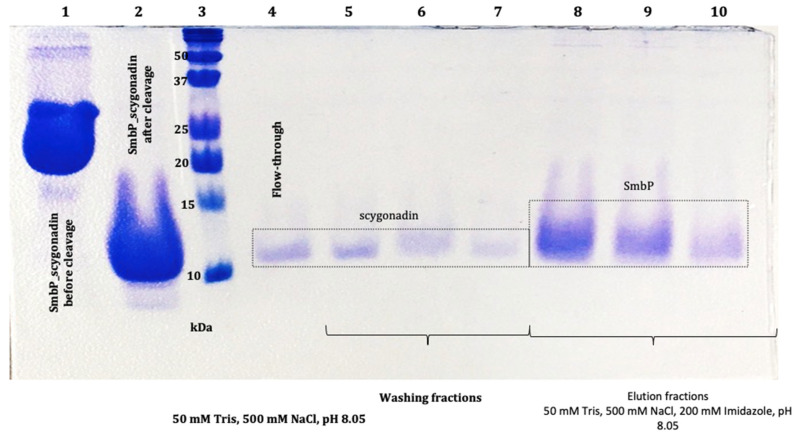
The 17% SDS-PAGE analysis of SmbP and SCY (9.86 kDa and 11.27 kDa, respectively) after enterokinase cleavage and tagged removal using a second round of IMAC: lane 1: SmbP_SCY (21.7 kDa) before enterokinase cleavage; lane 2: SmbP_SCY after enterokinase cleavage; lane 3: protein marker; lane 4: flow-through (SCY); lanes 5 to 7: washing fractions (SCY); lanes 8 to 10: elution fractions (SmbP).

**Figure 9:**
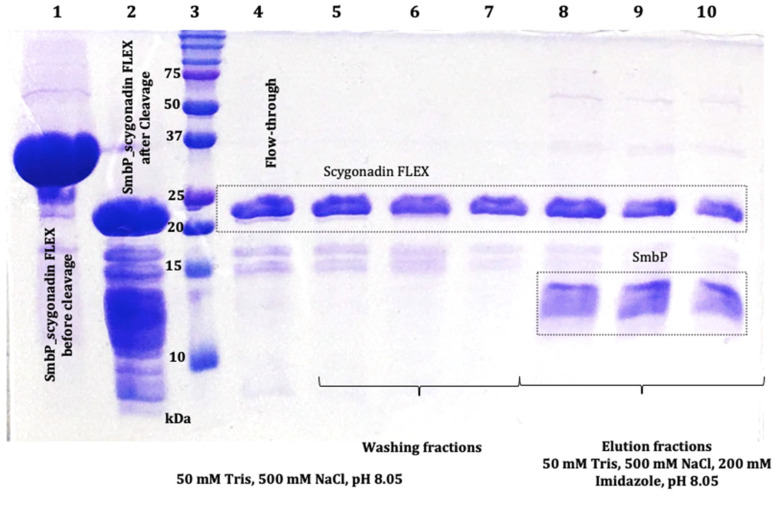
The 17% SDS-PAGE analysis of SmbP and SCY FLEX (9.86 kDa and 23.47 kDa, respectively) after enterokinase cleavage and tagged removal using IMAC; lane 1: SmbP_SCY FLEX (33.9 kDa) before enterokinase cleavage; lane 2: SmbP_SCY FLEX after enterokinase cleavage; lane 3: protein marker; lane 4: flow-through (SCY FLEX); lanes 5 to 7: washing fractions (SCY FLEX); lanes 8 to 10: elution fractions (SmbP and SCY FLEX).

Elution fractions were pooled and quantified using the BCA assay. The estimated protein concentration of SmbP_SCY was 4.04 mg/mL, giving a total of 38.38 mg per mL of IMAC column. The SmbP_SCY FLEX was 6.05 mg/mL, yielding 42.35 mg per mL of IMAC column. After purification, excess NaCl and imidazole were eliminated by dialysis against buffer 50 mM Tris-HCl, pH 7.4, followed by an enterokinase cleavage reaction, and SmbP was removed using a second round of IMAC. Figure 8 and Figure 9 show the electrophoretic analyses of these purification steps for both protein constructs.

### 3.3. Antimicrobial Activity

**Figure 10:**
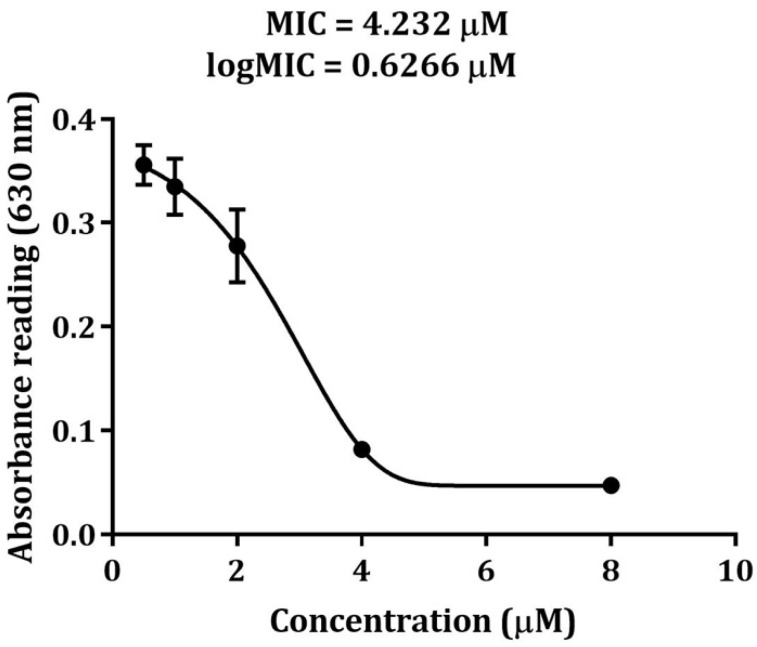
Antimicrobial activity of purified SCY against 1 × 10^5^ CFU/mL of *Staphylococcus aureus*. The data points represent the mean absorbance of each dose of SCY after 24 h of incubation from three replicas, and the error bars represent the standard deviation of the mean. The result of the Student’s *t*-test revealed significant differences compared to the negative control (*p* < 0.05).

Once SCY and SCY FLEX were released from the SmbP tag, a MIC assay was performed against *Staphylococcus aureus*. For recombinant SCY, a series of concentrations were tested (8.0, 4.0, 2.0, 1.0, and 0.5 μM); results showed a MIC of 4.23 μM, as illustrated in Figure 10. SCY FLEX was also assessed with the following concentrations: 40, 20, 10, 5, and 2.5 μM, showing an antibacterial effect on *S. aureus* at 40 μM.

## 4. Discussion

As with many other antimicrobial peptides isolated from mammals, bacteria, or fungi, it is practically impossible to extract enough quantities from the natural source; therefore, applying heterologous expression systems is a suitable strategy for the high-scale production of these needed peptides. Here, we employed SmbP as a carrier protein for the expression and purification of SCY in combination with the *E. coli* strain SHuffle T7 to obtain a fully active antimicrobial peptide. Our laboratory has described SmbP as an attractive fusion protein for recombinant protein production in *E. coli*. We have previously produced recombinant peptides and proteins with disulfide bonds tagged with SmbP. For example, we expressed the human growth hormone in *E. coli* BL21(DE3) using an engineered SmbP version containing the PelB signal sequence for protein translocation to the periplasm since disulfide bonds can be formed correctly on this site [24]. Alternatively, cationic antimicrobial peptides, like Bin1b, VpDef, and LL-37, have been expressed in the cytoplasm of *E. coli* SHuffle T7, where we confirmed the proper formation of disulfide bonds [25,26,27]. This specific strain of *E. coli* has mutations in the thioredoxin reductase (*trxB*) and glutathione reductase (*gor*) genes, which block the reductive pathways involving the enzymes allowing the formation of disulfide bonds. It also has the disulfide bond isomerase gene (*dsbC*), which is responsible for disulfide bond formation and proper folding. In this work, we describe for the first time the expression and purification of the anionic antimicrobial peptide scygonadin tagged with SmbP and the advantages this carrier protein represents.

SCY has been previously expressed in *E. coli* using the stain BL21(DE3) and the expression vector pTrc-CKS; it was also purified using IMAC, obtaining 97.5 mg per liter of cell culture [18]. Nevertheless, there is no mention of SCY containing disulfide bonds since BL21(DE3) cannot form them in its cytoplasm. Here, we obtained 38.38 mg of SmbP_SCY; even though it is less than previously reported, it is important to mention that we only used one 1 mL column, which can bind around 40 mg, therefore saturating it. The electrophoresis analysis showed the presence of SmbP_SCY in the flow-through; therefore, a larger column volume will retain more protein, increasing the total amount of protein. We can compare the amount of SmbP_SCY with the cationic antimicrobial peptides SmbP_Bin1b (4.4 mg/L), SmbP_VpDef (5.28 mg/L), and SmbP_LL-37 (3.6 mg/L) [25,26,27]. Our results showed we obtained almost ten times more protein than other antimicrobials produced; this difference might be because of the nature of each peptide [28,29].

The recombinant expression of proteins by multimeric tandems repeats a common strategy for expressing antimicrobial peptides; it consists of copying the antimicrobial gene several times to increase gene transcription and expression [13]. However, a higher number of repeats does not translate to an increase in the transcription and translation of the gene; it depends on each peptide [30,31,32]. Here, we report for the first time the expression of the multimeric tandem repeat of SCY; a total of 42.25 mg for SmbP_SCY FLEX were obtained (using only a 1 mL IMAC column), showing a slight increase compared to SmbP_SCY. As mentioned before, some codons have a longer translation time known as flexible unions or sequences; it is believed that these codons reduce the rate of protein synthesis, allowing the amino acids that are already part of the chain to have more time available for the protein to fold [33]. Since the best-known flexible sequence is (GGGGS)n, we used it and repeated it three times (Figure 2A). Here, we combined these systems by designing SCY FLEX, two identical encoding sequences for scygonadin linked with (GGGGS)_3_ as the flexible linker. This strategy has been applied to other antimicrobial peptides such as CM4 (3 repeats), mhBd2 (2 repeats), hPAB-β (3 repeats), and Bin1B (2 repeats), all of which are expressed in *E. coli* [31,34,35,36]. Our results showed that two repeats of SCY had been able to express in *E. coli* SHuffle T7 and were purified effectively.

We tested the antimicrobial activity of SCY and SCY FLEX against *S. aureus* since it is a member of the ESKAPE pathogens group (*Enterococcus faecium*, *Staphylococcus aureus*, *Klebsiella pneumoniae*, *Acinetobacter baumannii*, *Pseudomonas aeruginosa*, and *Enterobacter* species) and one of the Gram-positive bacteria associated with nosocomial infections, placing a significant burden on healthcare systems [37]. The MIC calculated for SCY was 4.23 μM; previous reports like pET28-scygonadin, also expressed in *E. coli*, showed activity between 7.5–15 μM against *S. aureus* [18]. Comparing it with our results, we have the same effect but at a lower minimum inhibitory concentration. Differences in peptide biological activity, particularly a reduced MIC, can manifest during the expression of peptides in *E. coli* [38]. Various factors may contribute to this result; the influence of the fusion partner on folding and stability is a crucial aspect, where deviations can lead to misfolding, aggregation, or proteolytic degradation, thereby impacting biological activity [39]. Complete proteolytic cleavage is also important since the partial separation of the target peptide from the fusion partner results in interference with the peptide function or masking of its binding site [40]. Downstream processing, including purification, can induce stress on the peptide, affecting its activity [41]. Therefore, optimizing these factors is essential for achieving higher yields and optimal activity in the desired peptide. The combination of SmbP as the carrier protein, *E. coli* SHuffle T7(DE3) as the expression host, IMAC as the purification method, and the use of enterokinase for tag removal and SCY separation produced an antimicrobial peptide with a lower MIC.

Our results for SCY FLEX showed no *S. aureus* growth at 40 μM, approximately ten times more concentration than SCY. Even though we obtained more protein after IMAC purification for SCY FLEX, we also observed that a higher concentration is needed for total antimicrobial activity. A higher minimum inhibitory concentration observed in a dimeric antimicrobial peptide in comparison to its monomeric counterpart can be attributed to several potential mechanisms [42]. The altered membrane interaction resulting from dimerization is a key factor, as the increased size of the dimer may impede effective penetration and disruption of the bacterial membrane, ultimately diminishing its antimicrobial efficacy. Additionally, the dimeric structure may shield crucial functional groups responsible for membrane interaction, such as hydrophobic or cationic residues, thereby reducing their binding affinity to the membrane [43]. There are no previous reports about SCY expression as a tandem repeat using flexible sequences and the effects on its antimicrobial activity. The results from this work might help others decide if the tandem repeat strategy is the proper method for antimicrobial peptide production since it increases the amount of protein but somewhat diminishes its activity.

## 5. Conclusions

This study demonstrates the advantages of using SmbP for expressing and purifying the antimicrobial peptide scygonadin and its multimeric tandem repeat. SmbP improves their soluble expression and purification, obtaining significant amounts of active antimicrobial peptides against *Staphylococcus aureus*. Furthermore, we reported the lowest minimum inhibitory concentration for scygonadin and its first tandem repeat expression strategy. Therefore, SmbP is a valuable carrier protein for the recombinant production of biologically active peptides in *E. coli*.

## Data Availability

All data supporting the findings of this study are available upon request.
